# Serotonin Syndrome with Monotherapy of Low-Dose Sertraline in an Adult Patient with Autism Spectrum Disorder

**DOI:** 10.1155/2023/6610152

**Published:** 2023-06-06

**Authors:** Rohit Madan, Jody Platto, Senthil Rajaram Manoharan, Varun Monga

**Affiliations:** ^1^University of Arizona College of Medicine, Tucson, Arizona, USA; ^2^University of Alabama in Huntsville, Huntsville, Alabama, USA; ^3^Banner Thunderbird Medical Center, Glendale, Arizona, USA

## Abstract

Serotonin syndrome, also known as serotonin toxicity, is associated with increased serotonergic activity in the central and the peripheral nervous system. The symptoms can range from mild to potentially life threatening. Given the widespread use of serotonergic agents, the number of cases is on the rise. It is seen with therapeutic medication use, inadvertent interactions between drugs, and intentional self-poisoning, but still known cases with monotherapy of selective serotonin reuptake inhibitors are uncommon. Another known fact is that elevated whole blood serotonin, or hyperserotonemia, is one of the first biomarkers identified in autism spectrum disorder and is present in more than 25% of affected children. We present a case of a 32-year-old male with a history of autism spectrum disorder and depressive disorder who presented to the emergency department with restless agitation, neuromuscular excitability, and autonomic instability. He had been prescribed sertraline 50 mg which he had taken daily as prescribed for 4 days. On the fourth day, he presented to the emergency department with diffuse muscle stiffness, upper extremity tremors, ocular clonus, and inducible ankle clonus. He was diagnosed with probable serotonin syndrome utilizing Hunter's criteria. Patient's symptoms resolved within 24 hours with intravenous fluids, lorazepam, and discontinuation of sertraline. This case highlights the importance of a high degree of clinical suspicion in patients even on monotherapy of selective serotonin reuptake inhibitors in therapeutic doses, especially in children and adults with autism spectrum disorder. Due to preexisting hyperserotonemia, they may be more susceptible to serotonin syndrome than the general population.

## 1. Introduction

Antidepressant use is increasing throughout the world. During 2015–2018 as per National Center for Health Statistics published in 2020, 13.2% of adults aged 18 overused antidepressant medications in the past 30 days. [1] Among antidepressants, selective serotonin reuptake inhibitors (SSRIs) have widespread use for several different psychiatric indications due to their relatively good tolerability and low toxicity profile. Serotonin syndrome (SS), also referred to as serotonin toxicity or serotonin toxidrome, is an adverse drug reaction characterized by an exaggerated increase in serotonergic activity in the central and peripheral nervous systems [2–5]. It comprises a constellation of signs and symptoms related to behavioral changes, neuromuscular excitability, and autonomic instability. These symptoms can occur in mild, severe, and potentially fatal forms, presumably, on the extent of increased serotonin [2–4, 6, 7]. The clinical diagnosis of SS is typically made using Hunter's criteria, which is widely considered to be the most accurate tool available, with a sensitivity of 84 percent and specificity of 97 percent when compared to the gold standard diagnosis by a medical toxicologist. It is less likely to miss early, mild, or subacute forms of SS [6, 8].

Severe toxicity usually occurs only with a combination of two or more serotonergic drugs (even when each is at a therapeutic dose), one of which is generally a monoamine oxidase inhibitor (MAOI) [2–4, 6]. Moderate toxicity has been reported with an overdose of a single drug and occasionally from increasing therapeutic doses [2–4, 6, 9]. The serotonin-norepinephrine reuptake inhibitors (SNRIs, e.g., desvenlafaxine and venlafaxine) are slightly more likely to cause SS compared with SSRIs but several-fold less likely compared with MAOI's coingestion [9]. The United States' Toxic Exposure Surveillance System consistently reports tens of thousands of exposures to SSRIs, many of which involve SS [10]. Its incidence is difficult to assess, but in a large case series of overdoses, moderate SS occurred in 15% of poisonings with SSRIs [9]. There is no existing literature on the incidence of SS in patients with monotherapy of SSRIs presenting with SS. Given SS presents on a spectrum of clinical symptoms and severity, it is not easily suspected in patients with monotherapy as those cases are usually of mild to moderate severity [2–4, 6, 9]. In addition, elevated whole blood serotonin level or hyperserotonaemia was the first biomarker identified in autism spectrum disorder (ASD) and is present in more than 25% of affected children [11]. It is unknown if this association makes these patients more susceptible to SS.

To date, there are only 9 documented cases of SS from SSRI monotherapy at a therapeutic dose and only 3 with sertraline monotherapy in therapeutic doses [12–20]. The purpose of this case report is addition to the increasing number of very few documented cases of SS with SSRI monotherapy and is one of the first reported case in adult patient with ASD.

## 2. Case Presentation

A 32-year-old male with a history of high-functioning ASD (living independently), depression, hypertension, and acid reflux disease was presented. Collateral information from the father confirmed a diagnosis of ASD that was based on detailed neuropsychological testing in childhood. His home medications included losartan 100 mg daily, famotidine 20 mg daily, and sertraline 50 mg daily. Patient shared that he was started on sertraline for his depressive symptoms 4 days ago, and he had taken his 4^th^ dose that morning. He denied any previous history of antidepressant medication use. On the fourth day of sertraline 50 mg dosing, he presented himself to the emergency department (ED) during evening hours with complaints of restlessness and diffuse muscle stiffness; he felt his legs were “stiff and heavy,” and he “couldn't really move them” and also described “toes retching turning inwards” with an unusual but not painful sensation. On physical examination, he was noted to have bilateral upper extremity tremors, ocular clonus with lateral eye movement, and inducible clonus in his right ankle. Cranial nerve exams II-XII were normal, and deep tendon reflexes were not commented upon, so it is unclear if they were examined or not. Vital signs are notable for a heart rate of 61, blood pressure of 164/94, and oxygen saturation of 98% at room air. Routine lab work showed a normal complete blood count (CBC) with differential comprehensive metabolic panel (CMP) that was unremarkable other than mild hypokalemia of 3.3 mEq/L. Urine drug screen was negative for any substances. Thyroid stimulating hormone level (TSH) and urinalysis were normal. Electrocardiogram (EKG) showed sinus rhythm with QTc of 403.

Patient was diagnosed with SS using Hunter's criteria given both inducible and ocular clonus, with restless agitation and tremor. Other diagnoses (anticholinergic toxicity, intoxication from sympathomimetic agents, withdrawal from sedative-hypnotic, thyroid storm, and acute extrapyramidal syndromes) were ruled out as the patient denied taking any other medications or antipsychotic, illicit substances, or supplements on the day of presentation to the ER or before. There are no known drug interactions with the use of sertraline with losartan and famotidine.

He received IV fluids with repletion of potassium for mild hypokalemia and IV lorazepam 1 mg with a resolution of symptoms by the next morning and was discharged home with the recommendation to stop taking sertraline. A score of 7 (probable) was obtained using the Naranjo algorithm—adverse drug reaction probability scale to assess for adverse drug reaction [21]. Similarly, the WHO-UMC causality assessment system also suggested a probable/likely adverse drug reaction [22].

Table 1 is adapted from Dunkley et al. [8].

## 3. Discussion

SS usually is only suspected in cases of overdoses and where SSRIs are being used in conjunction with other medications that lead to excess of serotonergic activity. To date, only 9 case reports of SSRI-induced SS have been reported in the literature when used as monotherapy in therapeutic dose. There are only three reported cases with sertraline monotherapy, and one was in a 9-year-old child with chronic behavioral problems, who had severe SS with a single dose of sertraline 50 mg [16]. Another case report is of a 36-year-old male who had been on sertraline 100 mg along with hydroxyzine and marijuana use and who presented about two to three months of being on sertraline with SS [19]. There is another case report titled “SS following low-dose sertraline” [20], but no abstract or accessible report could be found. The true incidence or risk factors that predispose a person to SS on SSRI monotherapy are unknown. Diagnosis can be challenging as these cases are usually mild to moderate severity [2–4, 6, 9] and are easily missed given overlapping symptoms with other syndromes like anticholinergic toxicity, intoxication from sympathomimetic agents, withdrawal from sedative-hypnotic (e.g., alcohol, benzodiazepine, clonidine, and baclofen), thyroid storm, and acute extrapyramidal syndromes (e.g., dystonic reaction). Our patient with diagnosed ASD in childhood is one of the first cases of SS in this population. It is interesting to note that 25% of patients with ASD have hyperserotonergic activity, which is considered as one of the earliest biomarkers in these patients [11]. This could likely be a risk factor for monotherapy-related SS in patients with comorbid ASD.

Although tantalizing clues suggest that changes in peripheral biomarkers are linked to alterations in brain function and behavior in individuals with ASD, the precise role of the serotonin system in the pathophysiology of ASD remains incompletely understood [11]. More studies are needed to understand if there is any association with ASD and the risk of SS with SSRI monotherapy. ASD poses an additional challenge in diagnosis given communication difficulties, repetitive motor movements, and behavioral issues [23] that can lead to easily missed diagnosis of mild to moderate SS since its diagnosis is solely based on history and clinical findings.

Treatment of mild to moderate cases of SS includes discontinuing the offending SSRI. Supportive care is provided, the goals being adequate patient sedation and normal vital signs. Standard interventions include oxygen, intravenous (IV) fluids, and continuous cardiac monitoring. Autonomic instability and hyperthermia require aggressive treatment. Sedation is recommended for the treatment of agitation. Antipyretic agents (e.g., acetaminophen) are ineffective and should not be used for SS. In patients who do not show improvement in agitation or vital signs with benzodiazepines and supportive care, treatment with cyproheptadine (5HT_2A_ antagonist), an antagonist at histamine and serotonin receptors, is recommended. The initial dose is 12 mg, followed by 2 mg every two hours until a clinical response is seen. Treatment with cyproheptadine is based on anecdote and experience since clinical trials are not available. SS often resolves within 24 hours of discontinuing the serotonergic agent and initiating care, but drugs with long half-lives or active metabolites may cause symptoms to persist [2–4, 24].

## 4. Conclusions

Cases of SS from SSRI monotherapy usually present with mild to moderate severity and, therefore, are not easily suspected. Hunter's criteria are considered to be the most accurate to diagnose early, mild, or subacute forms. This case report highlights the importance of SS even in patients on SSRI monotherapy taking therapeutic dosages. It particularly emphasizes the significance of keeping a high degree of suspicion for SS in ASD patients as some of them may have baseline hyperserotonergic state. Early diagnosis and discontinuation of the serotonergic agent along with supportive management is the most effective treatment of this syndrome.

## Figures and Tables

**Table 1:** Hunter's criteria met in our patient. Positive in our patient is in bold.

Hunter serotonin toxicity criteria
Presence of a serotonergic agent
(i) **Recent addition** (ii) Increase dose/overdose (iii) Interaction

Meet ONE of the following conditions
(i) Spontaneous clonus (ii) **Inducible clonus PLUS agitation** or diaphoresis (iii) **Ocular clonus PLUS agitation** or diaphoresis (iv) Tremor PLUS hyperreflexia (v) Hypertonia PLUS temperature above 38°C PLUS ocular clonus or inducible clonus

## Data Availability

The clinical data supporting this case report is from previously reported similar case reports and literature review, all of which have been cited.
